# A Review and Meta-Analysis of the Prevalence and Health Impact of Polycystic Ovary Syndrome Among Medical and Dental Students

**DOI:** 10.7759/cureus.40141

**Published:** 2023-06-08

**Authors:** Tamara Coffin, Jadzia Wray, Ramsagar Sah, Mary Maj, Reetuparna Nath, Shreya Nauhria, Sabyasachi Maity, Samal Nauhria

**Affiliations:** 1 Medicine, St. George’s University School of Medicine, St. George's, GRD; 2 Medicine, St. George's University School of Medicine, St. George's, GRD; 3 Biostatistics, Sagar Hospital, Bengaluru, IND; 4 Biochemistry, St. George's University School of Medicine, St. George's, GRD; 5 Educational Services, St. George's University, St. George's, GRD; 6 Psychology, University of Leicester, Leicester, GBR; 7 Physiology, St. George's University School of Medicine, St. George's, GRD; 8 Pathology, St. Matthew's University, Georgetown, CYM

**Keywords:** health education & awareness, systematic review and meta-analysis, prevalence rate, medical school students, polycystic ovary syndrome (pcos)

## Abstract

Women currently comprise the majority of students graduating from medical school and face unique stressors not seen with their male counterparts. In particular, during their medical education, women with polycystic ovary syndrome (PCOS) experience symptoms of the disorder, which strongly impact both their academic and social lives. This in turn influences their academic and professional future. Although women as medical professionals, in general, are happy with their careers, awareness, and understanding on the part of medical educators will definitely be helpful to women medical students in their path to being successful medical professionals. The first objective of our current study is to find the prevalence of PCOS in medical and dental students. The second objective is to find the academic and health impacts of PCOS and what type of interventions are being adopted to relieve the symptoms. Using the keywords such as “PCOS,” “medical students,” and “dental students”, the search engines PubMed, Embase, and Scopus were used to retrieve relevant articles published from 2020 to 2022. After removing the duplicates, 11 prospective cross-sectional studies were utilized for qualitative and quantitative analysis. The pooled prevalence of 2,206 female medical students diagnosed with PCOS was 24.7%. The students in the various studies were aware of their PCOS diagnosis and were taking therapeutic medications. The most common associated complications reported were BMI abnormalities, abnormal hair growth, and acne, along with other complications such as stress and poor academic and social life. The majority also had significant family histories of concurrent clinical conditions such as diabetes, hypertension, and other menstrual abnormalities. Considering the huge impact of PCOS, medical educators, policymakers, and all stakeholders should take proactive measures to accommodate students’ needs and bridge the social gap. Special awareness of needed lifestyle changes should be a part of the medical education curriculum for an inclusive educational environment, as it will minimize the gender discrepancy in academic satisfaction and professional life.

## Introduction and background

Polycystic ovary syndrome (PCOS) is a relatively common but often ignored endocrinological complication that affects nearly 116 million women worldwide. According to World Health Organization (WHO) studies in 2012, women of reproductive age with PCOS often experience clinical symptoms that lead to compromised academic and professional success [1]. The most prevalent clinical characteristics of PCOS are oligomenorrhoea, hirsutism, and acne. In addition, PCOS is also associated with other metabolic disorders including insulin resistance and increased expression of sex hormone levels. Therefore, women with PCOS have a higher risk of cardiovascular diseases (CVDs), cerebrovascular diseases, and metabolic syndromes. Women with PCOS have a higher prevalence of obesity, impaired glucose tolerance, and type II diabetes. Further, they display features of cardiometabolic syndrome, which include hypertension, dyslipidemia, visceral obesity, and insulin resistance [2,3]. A recent meta-analysis study comprising 166,682 subjects found an increased risk of CVDs and cerebrovascular disease among women with PCOS, including the occurrence of myocardial infarction, ischemic heart disease, and stroke [4]. PCOS during the period of pregnancy is associated with a significantly increased risk of adverse outcomes including gestational diabetes mellitus (GDM), preeclampsia, preterm delivery, cesarean delivery, miscarriage, hypoglycemia, and perinatal death. In 2016, researchers provided meta-analytic evidence, based on the study of 17,816 pregnancies of women with PCOS, that there is a significantly increased risk of adverse pregnancy, fetal, and neonatal outcomes [5]. A recent meta-analysis research found that women with PCOS are more prone to developing cancer of the endometrium (out of a total of 4056 women) and ovarian cancer (out of a total of 4,547 women), but no significant prevalence of breast cancer (out of 23,842 women) [6]. Overall, the quality of life (QOL) of women across the world is significantly reduced because of PCOS [7].

Numerous studies have shown that American and Canadian medical students in general experience a high rate of depression and anxiety. The overall psychological distress is consistently higher among female medical students compared to age-matched male peers [8]. A cross-sectional study of female medical students found that discomfort due to menstrual abnormalities led to a reduction in the quality of life (QOL) in approximately 50% of subjects [9]. A positive correlation was found between menstrual abnormality and stress-associated depression and anxiety in a study of 414 medical students [10]. A recent systematic review and meta-analysis further provided an extensive overview of the prevalence and impact of menstrual abnormality among female medical students worldwide [11]. Studies specific to women with PCOS show that they are struggling to cope with both the physiological and psychological effects of the disorder while studying in medical school [12-16]. Expression of the clinical features of PCOS has been shown to negatively impact women’s overall academic performances [17,18]. A recent systematic review outlines specific associations between PCOS-mediated menstrual abnormality and negative academic behavior such as increased absenteeism, lesser participation in classroom activities, lack of concentration, and poor academic performance [19]. Abnormal menstruation due to PCOS leading to academic absenteeism and reduced attendance at social events has been shown to further increase anxiety. These studies suggest that up to 20% of medical and dental students with PCOS experience severe pain [20-22], resulting in a reduced QOL, which then leads to symptoms of depression [23]. Targeted stress management programs have been shown to provide significant benefits in reducing depression and anxiety symptoms, leading to improvements in QOL among PCOS patients [24]. Alarmingly, other groups have shown that women with PCOS exhibiting associated complications are generally advised to take various painkillers and other drugs such as metformin, leading to life-long dependency on these pharmaceutical agents [25-28].

There are three main groups, which report variable diagnostic criteria of PCOS. They are the National Institute of Health (NIH), the European Society for Human Reproduction and Embryology (Rotterdam criteria) and the Androgen Excess and PCOS society. Due to these differing criteria, there are inconsistencies in the reporting of the prevalence of PCOS [29,30]. Irregular menstrual cycles are common in women of early reproductive age, which often confounds the diagnosis of PCOS till much later in adulthood, leading to a delay in appropriate therapeutic treatments and skewed prevalence data. Although a few systematic reviews addressed the prevalence of PCOS in the female population [31-33], it is difficult to correlate the published data due to the variable diagnostic criteria and age of onset.

With the recent positive attitude toward providing inclusive environments, most institutions for medical education have reformed policies to accommodate the needs of all students, including those with a broad range of disabilities [8,34]. Researchers have also reported that 2.7% of allopathic medical students have some sort of disability, including chronic physical and mental disabilities [35]. The majority of other studies show that slightly greater than 50% of medical students in America are female, most of whom are in their reproductive years. The NIH has estimated that 4%-20% of women in their reproductive age are affected by PCOS. Thus, female medical students who suffer the complications of PCOS appear to be underrepresented by most studies, leading to inadequate resources and lack of awareness of the particular needs of such students. Educators need to first understand female-centric health issues such as PCOS that have been misunderstood or misrepresented in the past. One way to achieve this is by eliminating the knowledge gap on the prevalence of PCOS and the particular health impact on the lives of the women affected [36]. We explore the prevalence of PCOS and discuss its impact on the physical, psychological, and academic characteristics of female medical students. We also highlight the intrinsic inequality of the medical school curriculum in considering the complexities of the common abnormalities of female physiology. This review also focuses on the perception or previous knowledge of PCOS in managing PCOS related complications.

Thus, the aims of the current study are to probe the prevalence of PCOS using meta-analysis and to assess the effect of this prevalence on health and overall QOL among medicine students. A secondary aim will be to provide an overview of the modes of interventions adopted by female students to reduce the clinical symptoms of menstrual disturbances.

## Review

Materials and methods

Protocol

This review was designed and reported in accordance with the Preferred Reporting Items for Systematic Reviews and Meta-Analyses (PRISMA) guidelines [37]. Ethical approval was not required. The protocol was registered in the International Prospective Register of Systematic Reviews (PROSPERO 2023 CRD42023399718).

Literature Search

Published studies were searched in electronic databases, namely PubMed (US National Library of Medicine, National Institutes of Health), Scopus, and Embase for potentially relevant studies from 2020 up to 2022. Articles published only in English from selected databases were included. The authors were required to reach a consensus among themselves on the final search strategy. The medical subject headings (MeSH) search terms included “PCOS,” “PCOD,” “dental student,” and “medical student,” including all subheadings. Similar database-specific search strategies with the same MeSH keywords were used to find the relevant articles in Embase and Scopus. Finally, the relevant articles were also included by adopting the snowball method of hand searching, which involves searching the bibliographic list of selected articles.

Selection of Studies

**Figure 1:**
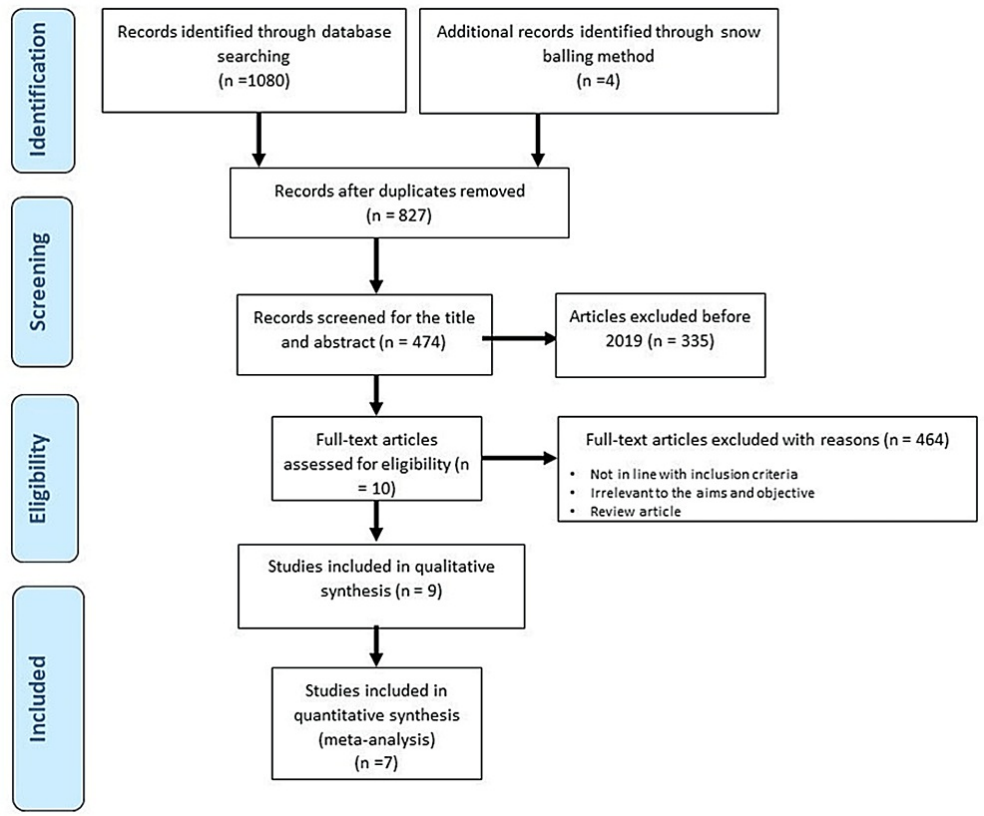
PRISMA protocol of literature search process

Two independent reviewers (TC and ZW) screened the retrieved papers based on titles and abstracts. The screening was validated by SM and SN independently. The criteria for examination of the full text of the relevant papers after the initial database screening were decided. Articles reporting data on the prevalence of PCOS that could be extracted for statistical analysis were included. The non-peer-reviewed editorials, letters, commentaries, incomplete data, reviews, conference posters, preprints, and theses were excluded. Any confusion or doubts regarding the study selection were resolved by reaching a consensus. Figure 1 represents the process of study selection for the systematic review and meta-analysis as per the PRISMA protocol.

Data Extraction

Author SM extracted the relevant data, and the data was crosschecked by SaN and others. In a blank Excel sheet, data on author and year of publication, geographical location, type of study, and relevant qualitative and quantitative data for each eligible study were extracted. Any disagreement among authors was resolved by consensus.

Data Analysis

A meta-analysis of quantitative data was performed to estimate the cumulative prevalence from individual studies. The summary estimates of prevalence were reported along with their 95% confidence intervals (CI) for PCOS among female medical and dental students. The pooled prevalence data was presented in a Forest Plot. All the analyses were done using the “Comprehensive meta-analysis” software version 3. The presence of publication bias was not examined since the included articles for meta-analysis were less than 10 in number.

Results

Search Results and Study Characteristics

Our search through the databases finally identified 10 articles on the prevalence, knowledge, and impact of PCOS that were included in the systematic review. The article exclusion criteria were used to remove articles irrelevant to the stated aims and objectives. Studies conducted on subjects other than medical and dental students were not included. Articles where full text files were not available were also removed.

All the included studies were of cross-sectional and perception survey types across the world at varied numbers and time frames. The total sum of female medical students included in the selected studies was 2,206. These studies reported on the prevalence of PCOS, the health impact of PCOS either as major or as minor complications, the previous knowledge of PCOS, and the major risk factors. In addition, some studies reported the family history of other diseases and any interventions adopted by the students to alleviate the psychosomatic discomfort of PCOS. The prevalence data were calculated accordingly using the meta-analysis protocol. A detailed synthesis and summary of the included studies are provided in Table 1.

**Table 1:** Qualitative synthesis of 10 included studies

Author, year, and location	Study type	Age group	Medical/Dental (n)	Prevalence of PCOS	Diagnostic criteria/Assessment tool	Conclusion
Shreeyanta, 2020, Nepal [14]	Descriptive cross-sectional study	20–29 years	Medical, n = 381	9.18% of students had PCOS	A self-administered closed-ended questionnaire for the diagnosis of PCOS by Pedersen SD, et al. 2007 was used for the screening of PCOS	This study concluded that PCOS remained a growing endocrinological difficulty among females in their reproductive years. Screening from early age is essential to intercept lifelong complications.
George, 2020, India [15]	Cross-sectional survey	18–25 years	Dental, n = 100	Not reported adequately	Questionnaire-based survey	This study highlighted an increasing need for the recognition of PCOS among female dentists. When diagnosed and treated early, there is an improvement in the quality of life and an increased ability to avoid future health hazards.
Swetha, 2019, India [38]	Cross-sectional study	18–22	Medical, n = 113	The prevalence rate of PCOS among obese females was 85.8%	Rotterdam criteria	This study reported a PCOS prevalence rate of 85.8% among obese females, indicating an association between the two factors. The study also concluded that an increase in BMI causes an increase in the risk of PCOS in females.
Sogasu, 2019, India [39]	Survey	Women of reproductive age (not specified)	Dental, n = 100	42% have PCOS	Questionnaire-based survey	This study emphasized PCOS-associated challenges faced by women and girls of reproductive age in today’s society. Diagnosing and intervening early on can reduce the side effects of the condition.
Aggarwal, 2019, India [40]	Cross-sectional study	17–24 years	A total of 456 medical, dental, and physiotherapy students. 91.67% were studying MBBS	21.05% of students had been diagnosed with PCOS	A detailed, self-administered questionnaire was prepared on the basis of the Cronin et al. (1998) questionnaire.	This study reported PCOS as a common and growing condition among young women that requires lifestyle modification, greater awareness, and early diagnosis to thwart further impediments.
Tahir, 2020, Pakistan [41]	Cross-sectional type of quantitative study	17–30 years	278 individuals, according to the WHO formula. Out of the 278 individuals, 94.2% were female medical students, and the remainder were female dental students of CMH LMDC.	11.2% of the students were diagnosed with PCOS	Questionnaire-based survey. PCOS was diagnosed clinically	Results of this study indicate that although 74.5% of female medical students were aware of PCOS, at most 33.8% of the students had a normal 28-day menstrual cycle. Additionally, the study reported associations between PCOS and the duration of menstrual cycles, BMI, and symptoms such as weight gain and acne.
Ahmad, 2020, Pakistan [42]	Cross-sectional study	The mean age of 21 ± 1.6years	A total of 242 students were included in the study with a mean age of 21 ± 1.6 years	The frequency of PCOS was 19.4%	Rotterdam Consensus Criteria for PCOS	This study reported a high frequency of PCOS in female medical students. The study also highlighted a need for a national study to quantify the prevalence of PCOS in the young female population because of the potential for long-term ramifications on reproductive and metabolic health.
Subhashree BM, 2021, India [43]	Cross-sectional study	Not mentioned	192 participants were included in the study with 40.62% (78) being male and 58.95% (112) being female	Not reported adequately	A pretested semi-structured questionnaire was devised and validated their knowledge and awareness about PCOS.	This study highlighted the importance of educating people on ways to prevent and diagnose PCOS, in order to make the appropriate lifestyle changes to deter future complications.
Fattah, 2021, Iran [44]	Cross-sectional descriptive study	15–45 years	n = 636 (medical)	A total of 73 patients (11.5%) had PCOS	The PCOS screening questionnaire was designed by Pedersen et al. in 2007.	The study demonstrated a significant relationship between waist circumference (WC) and the prevalence of PCOS. For every 1 cm increase in WC, the odds ratio of the occurrence of PCOS increases by 7%.

**Figure 2:**
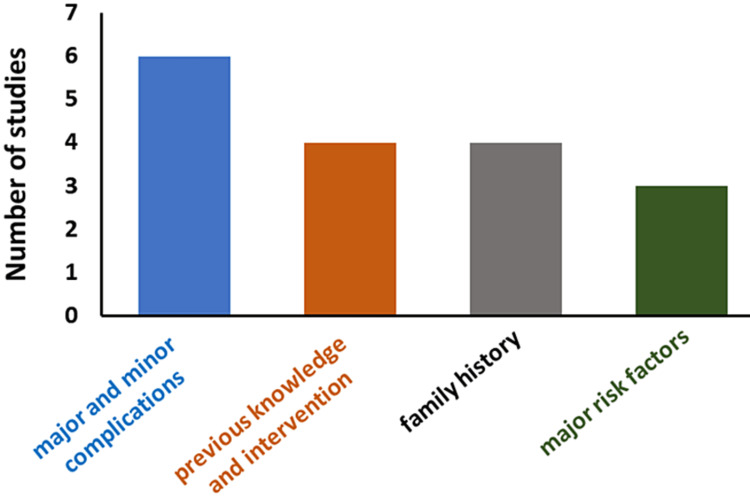
Four aspects of PCOS for the included studies

Figure 2 depicts the four primary aspects of PCOS among medical students such as health impact, major risk factors, family history, and previous knowledge of PCOS. A further breakdown of each of these aspects are explained below.

Psychosomatic Impact of PCOS

Patients of PCOS experience several clinical complications including menstrual disturbances, metabolic and CVDs, skin and hair problems, mental depression, and endometrial cancer. A recent systematic review summarizes these complications of PCOS among women of reproductive‑age [31]. Similarly, the major complications of menstruation abnormalities, hirsutism, and obesity were also observed from our included studies. Other complications include skin pigmentation and acne, mental depression, and alopecia. The pooled data from recent reports analyzed here concurs with older systematic reviews of the complications of PCOS [45].

Six studies discussed the health impact of PCOS. A detailed analysis of the health impact of PCOS is given in Table 2. The common themes of the adverse health impact of PCOS include menstrual disturbances, hirsutism, obesity/overweight, and other miscellaneous complications.

**Table 2:** Health impact of PCOS

Author and Year	Major complications			Other complications
	Menstrual disturbances	Hirsutism	Obesity/overweight	
Shreeyanta KC, 2020 [14]	21% participants reported prolonged (34 days) or variable menstrual cycles.	7.34% participants reported growth of dark, coarse hair in 3+ sites on their bodies.	21% participants reported being classified as obese or overweight.	1.049% participants had nipple discharge of a milky consistency.
Swetha T, 2019 [38]	Not discussed	59.2% students reported to have PCOS-associated facial hair growth.	The study included participants with BMI of more than 25. The mean BMI reported was 28.36 + 4.27 kg/m^2^.	57.5% patients had acanthosis nigricans
Sogasu D, 2019 [39]	44% reported heavy menstrual bleeding	Not discussed	Not discussed	51% of patients experienced anemia
Aggarwal M, 2019 [40]	90% of the students experienced irregular menstrual cycles and cramps.	The study found a significant association (p<0.05) between PCOS and hirsutism. 75% of the women with PCOS reported hirsutism and emotional problems.	In the high-risk group, 62.5% were either obese or overweight while in the low-risk group, 70% were either obese or overweight.	21% noted skin hyperpigmentation to be signiﬁcantly associated with PCOS. 55.4% of the students reported oily skin and acne. Students in the high-risk group experienced emotional problems like moodiness and were easily fatigued.
Tahir H, 2020 [41]	25% of the participants mentioned a cycle of 26 days. 7.9% reported menstrual cycle lasting 25 days or less. 1.4% had monthly menstrual cycle of 30 or more days. 37.1% students suffered from metrorrhagia or amenorrhea.	50.7% of students reported to have excessive facial or body hair	36.3% encountered weight gain	55.4% experienced oily skin and acne, while 42.8% suffered from alopecia.
Ahmad M, 2020 [42]	32% students reported symptoms of irregular periods	32% students reported excessive body hair	13% students had BMI>30	5% students suffered from thyroid dysfunction, 2% experienced hyperprolactinemia

Prevalence of PCOS

**Figure 3:**
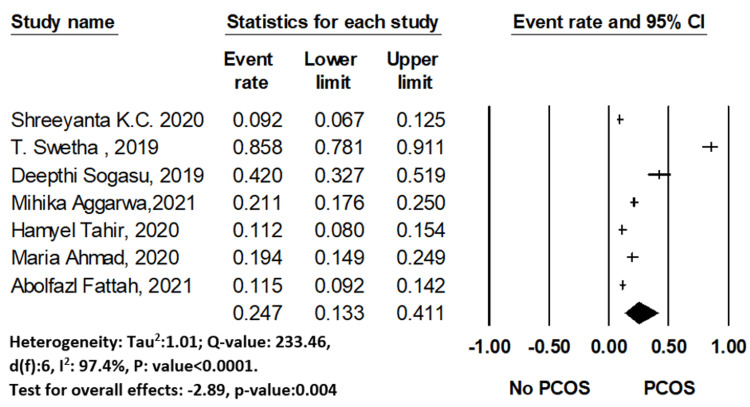
The forest plot of PCOS prevalence. The diamond represents the overall results and 95% confidence interval of the random effect of the meta-analysis. Model: Random, overall effect size: 0.247, 95%CI: [0.133-0.411], I 2 :97.4. Tau2 : 1.01. Shreeyanta [14], Swetha [38], Sogasu [39], Aggarwal [40], Tahir [41], Ahmad [42], Fattah [44]

Among the total female medical students of the included studies (n = 2,498), the prevalence of PCOS was reported by seven studies (n = 2,206). The pooled prevalence of PCOS was 24.7% (95% CI: 0.133-0.411). The studies of PCOS prevalence were conducted mostly in Asia. Figure 3 shows the overall PCOS prevalence with a high level of heterogeneity. The highest prevalence of PCOS was found from India [38], estimated at 85.80% (95% CI: 0.781-0.911) and lowest was also from India [14] at 9.2% (95% CI: 0.067-0.125). This observation of prevalence is higher than the previous observed studies in the general population [45] indicating the medical students have additional risk factors triggering PCOS.

Major Risk Factors and Medical History of Family

Among the included articles, three studies indicated major risk factors associated with PCOS and four studies indicated previous medical history in the family among the students [14,40-42,44]. The risk factors included preexisting obesity, endocrine issues such as hypothyroidism, mental issues such as anxiety or mood disorders, anorexia or bulimia, hypertension, diabetes, alcohol intake and smoking, lack of physical exercise, and frequent use of contraceptives. The students from these reports indicated a family history of chronic metabolic and CVDs such as type 2 diabetes mellitus and hypertension.

In addition, for every one cm increase in waist circumference, the odds ratio of developing PCOS increased by 8% [44]. The odds ratio of developing PCOS was 3.22 times higher in people taking contraceptives and hormonal medication than people taking no medication as reported by the same study. Four of the included studies also reported the past medical history of the family with PCOS [14,40,41]. Positive family history of PCOS was identified in 2.9%, 58.3%, and 12.6% of the students in three out of four studies. In addition, these studies also mentioned other medical conditions in the family-CVD (36.5%), diabetes (47.8%), carcinoma (7.9%), and irregular menstruation (21.3%). Participants’ mothers also had irregular menstruation.

Previous Knowledge and Lifestyle Factors Associated With PCOS

Knowledge and awareness regarding the prevalence of any disease provide proactive measures to either prevent the occurrence or manage the disease better. Although many students had previous understanding of PCOS, others either did not know about the disease or were not able to adjust lifestyle accordingly to prevent its occurrence. The lack of regular exercise or physical activity and poor diet were also found for this group of students. Our analysis found four studies that discussed this aspect.

A number of recent studies hypothesize that the early detection and implementation of therapy for PCOS would lead to improved outcomes of long-term effects associated with this disorder. Certain lifestyle choices are thought to predispose one to PCOS, which can be changed if females have a general knowledge of the signs and symptoms of PCOS. In a survey report of 100 female dental students showed that the majority of the females who were familiar with the term PCOS were also familiar with the symptoms, causes, and consequences of PCOS [15]. 61.9% of the females surveyed were unfamiliar with the development of PCOS. The participants were asked which clinical signs might lead to an increased chance of developing PCOS. Irregular menstrual cycles are the most common symptom of PCOS and 71.7% of the females who were surveyed believed that this increased a female’s chance of developing PCOS whereas 61.9% believed that a significant increase in androgen levels increased the chances for PCOS. Next, the females were asked about the common complications of PCOS if left untreated. 73.7% believed that uterine cancers and difficulty in conceiving were the most common complications of PCOS treatment that were neglected. For the underlying causes of the syndrome, 71.4% believed genetics, diabetes, and obesity to be contributing factors. Lastly, the most threatening effects of PCOS was perceived to be infertility, sleep apnea, and depression.

Two recent studies were conducted to identify the level of awareness that female students had PCOS. In 2020, a survey conducted on 278 students reported that 74.5% were aware of PCOS and 42.2% reported that they learned about the disease through social media and other internet sources [41]. A similar study in 2021 surveyed 192 females to identify their knowledge of PCOS. They found that 57.8% had a good knowledge of the disorder, 36.9% had a fair knowledge, and 5.2% had a poor knowledge of PCOS [43].

In 2020, a survey of 242 female medical students found that 47 of those surveyed fit the Rotterdam criteria for PCOS [42]. These 47 students were then surveyed to describe their lifestyle choices and diet. 19% of them did not engage in any physical activity, 17% had a daily exercise regime, and the remainder exercised one to three times per week. Out of those that exercised, 22% walked and/or jogged, 13% went to the gym, and the rest swam or did yoga. For the 68% of the students who reported physical activity, the duration ranged from 15 to 60 minutes. Food choices were also surveyed. 78% reported using fats/oils/sweets once or more daily; 64% ate fruits and vegetables once or more daily; 68% consumed dairy every day; 87% ate legumes once per day or per week; 81% ate meat once or more per day; and 94% consumed chapatti/bread/rice/pasta once or more per day. Fast food consumption among students with PCOS was also recorded where 12% of the students consumed it more than once per day, 17% once daily, 60% weekly, and 11% consumed it monthly.

Various Interventions by Students to Alleviate PCOS Complications

Many women needed to adopt a combination of lifestyle changes and pharmacological interventions to treat their PCOS symptoms. Pharmacological intervention by medical students was common in reducing the symptoms of PCOS. Most of the students reported self-medication by using over-the-counter painkillers such as non-steroidal anti-inflammatory drugs (NSAIDs) and other medications such as metformin.

Three of the four studies that reported pharmacological intervention mentioned the usage of metformin alone or in combination with oral contraceptive pills [40-42]. One study reported 52% usage of iron supplements for anemia associated with PCOS and 56% of the students with PCOS demonstrated an improvement in Hb levels following the use of iron supplements [39]. The usage of herbal medication was prevalent in one study [41]. Interestingly, 76.6%, 29.1% and 19.4% students with PCOS in two separate studies also indicated not taking any treatment to alleviate the complications [40-42].

Discussion

This study explores meta-analytic evidence of the prevalence and health impact of PCOS for affected medical students. A comprehensive search for recent articles (2019-2022), which reported relevant data for female medical students with PCOS, was performed and relevant data was extracted and summarized. Among a total of 2498 female medical students included in this meta-analysis and systematic review, PCOS prevalence was 24.7% based on the various criteria including Rotterdam criteria.

Overlapping psychosomatic events of puberty in PCOS pose a challenge for diagnosis during adolescence but are included in the diagnostic criteria for adult women [46]. There was no uniformity in selecting the diagnostic criteria for the confirmation of PCOS among the medical students. The most prevalent PCOS diagnostic criteria are by the Rotterdam scale. However, a few articles used an established method based on the clinical diagnostic questionnaire [47]. This questionnaire included the prevalence of several PCOS clinical symptoms along with scores. For example, positive scoring is allotted for prolonged menses, hirsutism, and obesity. A milky discharge from the nipple may indicate other endocrine abnormalities similar to PCOS symptoms. If the participants answer yes to the milky discharge, then a negative score is given towards the calculation of the final score. Finally, if the total score ≥ 2 is consistent with the diagnosis of PCOS and < 2, is not consistent with the diagnosis of PCOS. None of the studies provided ultrasound evidence of PCOS. Additionally, self-administered questionnaire on weight, hirsutism, menstrual irregularity, emotional problems like feeling moody and easily fatigued, acne, and brownish/blackish discoloration of skin was also used by one article [48]. The questions were scored on a seven-point Likert scale, in which the normal level of functioning was scored as one and deviation from normal functioning was scored from two to seven. A mean score of ≥ 3.71 is considered a high risk for PCOS and < 3.71 is considered a low risk for PCOS.

PCOS is characterized by elevated plasma androgen levels, abnormal menstrual bleeding, and/or cysts on ovaries [49]. As per a report by the National Institutes of Health Office of Disease Prevention (NIH ODP), the annual healthcare expenditure for the identification and management of PCOS is $4 billion [50].

Pathophysiology of PCOS

From menarche to menopause, women of reproductive age are predominantly susceptible to psychosomatic changes leading to physical and mental stress due to the hormonal changes associated with the menstrual cycle. These biochemical fluctuations of different sex hormones further alter the hypothalamic-pituitary-gonadal/adrenal (HPG/HPA) axis to induce abnormal physiology leading to the imbalance of homeostatic functions. Although recent studies provide evidence of imbalances of other endocrine functions including high insulin levels and a deregulated anti-Müllerian hormone (AMH) in PCOS [51-53], the primary defect of the ovarian physiology due to the HPG/HPA abnormality plays a central role. A detailed role of different genomic and non-genomic factors leading to the pathophysiology of PCOS is out of scope here. However, an extensive review of this aspect is provided somewhere else [54,55].

Insulin resistance in obesity increases androgen concentration in the blood causing anovulatory menstruation [56]. Elevation of plasma androgen inhibits follicular development and causes the formation of cysts in the ovaries, anovulatory menstrual cycles, or abnormal endometrial changes [57]. Therefore, the arrest of follicular maturation indicates an ovarian abnormality. Likewise, a majority of the females with PCOS show symptoms of excess androgen such as hirsutism. Excess androgen production by the adrenal or ovary, inhibits the sex hormone-binding globulin (SHBG) concentrations, leading to increased free testosterone concentrations [58]. Indeed, elevated, free testosterone has been shown to contribute to PCOS. Another consequence of androgen excess is an increase in the number of growing follicles in PCOS leading to a classic ovarian phenotype of enlarged ovaries in PCOS. The clinical manifestation of dysregulated HPG/HPA axis in PCOS is observed as increased gonadotropin-releasing hormone (GnRH) secretion, which increases the luteinizing hormone (LH) release. Interestingly, follicular-stimulating hormone (FSH) levels are reduced. Exogenous administration of FSH, therefore, corrects the follicular arrest [59].

PCOS Among Medical and Dental Students

According to the recent studies summarized in this report, the prevalence of PCOS was found to be 24.7% (Figure 3), which is significantly higher than the estimated global prevalence of PCOS of 6%-7%. These results strongly suggest that the needs of female medical students must be recognized and addressed by medical institutions. Weather women with PCOS are to be considered having a disability is beyond the scope of this report, consideration of gender-based difficulties resulting in poor performance in medical school and a reduced QOL must be considered by school administrators. This should be applied not only to women in medical schools but to women from all walks of life. There are reports, which study the prevalence of PCOS in non-medical students. For example, researchers found that 9.13% of female students taking health sciences in South India were diagnosed with PCOS [29]. Similarly, researchers showed that the prevalence of Omani women having PCOS was 7% in a hospital-based study [60]. A study of young women in Bhopal, India found the prevalence of PCOS to be 8.2% [61]. The prevalence of PCOS among the Kashmiri women of India was reported to be dramatically higher, at 46.4% [62]. Similarly, high prevalence (32.11%) of PCOS was also noticed in another study among the students of a tertiary care teaching hospital [63].

The wide ranges reported for the prevalence of PCOS likely result from the lack of worldwide consolidated assessment criteria. For example, researchers have provided meta-analysis evidence to show that the overall prevalence and phenotype of PCOS are 6% and 10% according to the NIH criteria and Rotterdam or AE-PCOS Society criteria, respectively [45]. Additionally, changes in lifestyle, diet, and stress levels among medical students were shown to increase menstrual abnormalities [11]. A study in South Asia suggested that the urban lifestyle of young women caused endocrine disorders [64]. Among the 2,206 female medical students in our study, the high percentage of women with PCOS may reflect underlying stressors that may impact health. Our study, however, cannot rule out selection bias. For example, previous research indicated that factors such as different ethnic groups, the severity of PCOS presentation, and the prevalence of existing obesity may influence the phenotype of PCOS presentation [65]. The recently published studies summarized in this report represent medical students from different socioeconomic statuses with diverse lifestyles, familial risk factors, and diverse cultural backgrounds. Researchers found a strong correlation between women with PCOS and a family history of type-2 diabetes mellitus of 58.3% [40]. Additionally, studies showed a low correlation with typically reported major risk factors (9.18%) such as obesity, hirsutism, acne, hypothyroidism, anxiety, and anorexia/bulimia, although, most reported hypertension, smoking, lack of exercise, constant mood disorder and migraine [14]. The disparities between historical data and current reports studied may indicate selection bias or differences in methods for determining PCOS in patients.

As commonly reported for PCOS patients, we also found three major complications of hyperandrogenism, hyperinsulinemia, and adiposity (Table 2). In addition, other complications such as abnormal milky discharge from the nipple, hyperpigmentation, acanthosis nigricans (AN), and alopecia were reported. The medical basis for these clinical presentations is likely a result of excessive androgen-preventing ovulation. Normal shedding regrowth of endometrium does not occur, leading to the endometrium becoming thicker/shedding irregularly resulting in menstrual abnormalities such as heavy and/or prolonged bleeding with long-term menstrual abnormalities increasing the risk of endometrial cancer. Our analysis found menstrual irregularities among medical and dental students’ studies. For example, previous research reported heavy menses, irregular menstrual cycles and menstrual cramps among 90% of the PCOS students [39,40]. Another research study indicated prolonged menstrual cycle (more than 34 days) in 20% of participants [14]. Other studies have also highlighted the long-term consequence of PCOS as women diagnosed with the syndrome are more likely to develop cancer of the endometrium and ovarian cancer [66].

PCOS patients may also present with hirsutism throughout the body. The increased presence of male sex hormones such as testosterone, dihydrotestosterone, dehydroepiandrosterone sulfate (DHEAS), and androstenedione cause the growth and maturation of sexual hair. Typically, the high concentration of androgen in men compared to women, especially during and after puberty, causes the production of larger, curlier, and darker terminal hairs on sex-specific areas of the body [67]. Therefore, hirsutism in women with PCOS due to excess androgen production leads to excessive growth of terminal hair in the body [68]. Moreover, hyperandrogenemia in PCOS can also cause acne formation and alopecia [69]. The hair follicles themselves have differences in sensitivity against excess androgen in PCOS, leading to differences in the severity of hirsutism at a given level of androgen excess for different women with PCOS [70]. The Ferriman-Gallwey (FG) scoring system has been widely used in clinical practice to visually inspect the body hair pattern and assign a score to excessive terminal hair [71,72], leading to the evaluation of hirsutism and facilitating data comparison between women with PCOS and healthy women. The modified FG (mFG) score, from zero (no terminal hair) to four (male pattern hair) is traditionally used to evaluate hair growth in nine body areas for PCOS-associated hirsutism. Several factors including ethnic variations, skin type, and other factors such as obesity and insulin resistance should be considered while assessing hirsutism in PCOS, as these factors play an important role in a universal mFG score [73].

Obesity and insulin resistance are also known to be predisposing factors that trigger the expression of PCOS, though this may vary with different ethnic backgrounds [74]. Therefore, the ethnic origin should be considered when evaluating the mFG scores in different populations as per the international evidence-based guideline for PCOS assessment and management [75]. A systematic review compared hirsutism in women with PCOS from populations of different ethnicities and showed that compared to white women, East Asian women had less hirsutism (low mFG score), whereas Hispanic women, South Asian women, and Middle Eastern women had more hirsutism (high mFG score) [76]. Moreover, there was a significant association of mFG score with androstenedione (A4) and DHEAS from a total of 6,593 PCOS patients distributed across different countries [77]. Using the Rotterdam criteria, US NIH or Androgen Excess Society (AES) criteria, another recent systematic review provided a comprehensive distribution of hirsutism scores in women with PCOS, according to geographic locations [78]. Similar to the different studies, our studies found hirsutism to be a major complaint among medical and dental students. For example, almost 7.34% of the students were said to tend to grow dark, coarse hair in three or more body sites as per a study in Nepal [14]. 59.2% of the medical students had a positive association between PCOS and hirsutism in another study in India [38]. Another study from India showed that 75% of students with PCOS complained of hirsutism [40]. Similarly, in another study conducted in Pakistan, 50.7% of the medical students had hirsutism [41]. These examples in our study resonate with the ethnic and regional variation of androgen sensitivity for the expression of PCOS among medical and dental students.

Metabolic abnormalities in PCOS include defects in insulin action and β-cell function, leading to an increased risk for glucose intolerance and type 2 diabetes mellitus. Insulin resistance in PCOS further increases the cardiometabolic risks, which are greatly amplified again by obesity [79,80]. Due to the intricate relationship between PCOS-related metabolic disorders, the pathogenesis and management of PCOS become challenging. Does PCOS cause obesity or obesity causes PCOS? Interestingly, obesity and PCOS influence each other [81]. Therefore, pre-existing obesity is a risk factor for PCOS. However, PCOS causes further metabolic dysregulation leading to weight gain and prevents the efforts to establish effective weight-loss among PCOS patients. For simplicity, we will only briefly explain the role of PCOS in the expression of obesity and weight gain.

Epidemiological data has faithfully established the association between PCOS and obesity [82]. Metabolic processes in our body are the source of energy gain and expenditure, providing physiologic Basal Metabolic Rate (BMR). Therefore, an abnormal metabolism in PCOS and a decrease in energy expenditure could have a major role in developing obesity in PCOS. Indeed, metabolic heat production after a meal was significantly lower in women with PCOS compared to the control group as measured by indirect calorimetry in a study [83]. Interestingly, the resting metabolic rate-induced energy expenditure is similar in both of these groups. A similar result of equal resting metabolic rates between women with PCOS and women who are control subjects was found in another recent study too [84]. Since postprandial thermogenesis contributes a small amount of energy towards the metabolic processes, only a lack of energy expenditure cannot completely explain obesity in PCOS. Additionally, androgen-mediated inactivation of lipolysis at the subcutaneous tissue may contribute to weight gain in PCOS [85]. Our analysis found reports of obesity or weight gain associated with PCOS in female medical and dental students. For example, 20.73% of the participants reported being obese or overweight now or sometime in the past in a study in Nepal [14]. A study in India found that 62.5% of the students with PCOS were either obese or overweight [40]. A study in Pakistan found that 36.3% of the students with PCOS also encountered weight gain [41]. Our analysis is in line with a systematic review that found a significant association between obesity and insulin resistance in Indian women with PCOS [86]. Therefore, medical and dental students should be encouraged to pay attention to early symptoms of irregular menstrual cycles and adiposity and receive medical guidance to facilitate early diagnosis and treatment and to prevent co-morbidities associated with PCOS. Our studies also found other non-major complications of PCOS such as milky discharge from the nipples, hyperpigmentation of the skin, mental health issues, and alopecia. 51% of dental students with PCOS were affected by anemia in one study [39]. AN is an important diagnostic marker in identifying patients with an increased risk for type 2 diabetes mellitus among PCOS cases [87]. Interestingly, one of our studies found that more than half of the medical students in the study with PCOS had AN [38].

As discussed previously, PCOS increases the risk of other metabolic diseases leading to weight gain or obesity. However, other risk factors also increase the risk of PCOS. Pre-existing obesity, for example, has close links with the development of PCOS. One of the oldest studies done in the Northern Finland Birth Cohort (NFBC) in 1966 associated BMI and features of PCOS at all ages [82]. Although the mechanisms of insulin resistance in PCOS are yet to be explored, almost 50%-90% of women with PCOS show this biochemical feature [88,89]. It is, however, hypothesized that testosterone and the CAG nucleotide repeats within the androgen receptors contribute to insulin resistance [90]. The deficiency of the downstream signaling mechanism of insulin, which is mediated by the phosphatidylinositol 3-kinase (PI3-kinase) pathway has also been implicated in PCOS [79]. Insulin resistance causes compensatory hyperinsulinemia that further stimulates steroidogenesis [91]. In addition to excess androgen synthesis, hyperinsulinemia leads to ovulatory dysfunction and the stimulation of adrenal P450c17α activity. Weight gain due to any reason can further worsen insulin resistance and the features of the metabolic syndrome associated with PCOS. Therefore, the effects of weight-gain on insulin resistance and compensatory hyperinsulinemia, increased steroidogenesis, impaired PI3-kinase pathway, etc., contribute to PCOS pathogenesis and are therefore risk factors. Our study found several risk factors associated with PCOS. For example, in one study among 381 medical students, 10.5% had obesity, 3.7% had hirsutism, 44.1% had acne, 1.8% had hypothyroidism, 53.5% had anxiety, and 8.9% had anorexia/bulimia [14]. Similarly, hypertension was reported in 1.6% of the participants, diabetes in 0.5%, alcohol intake in 31.49%, smoking in 1.8%, and mood swings in 56.2%, whereas 53.3% lacked physical exercise in the same study. Migraine was present in 8.9% of the students with PCOS. In addition, the probability of developing PCOS in women taking regular contraceptives is 3.22 times higher than in women taking no such medications [41]. Therefore, it further proves that these risk factors could also increase the susceptibility to PCOS among medical and dental students.

Genetic and epigenetic factors from the family history also influence the development of PCOS in women [92]. Therefore, PCOS could also be inherited from family members as a major risk factor. Researchers have hypothesized that PCOS could be due to the autosomal dominant inheritance of some genes [93]. Indeed, PCOS is prevalent in nearly 55%-60% of several small families of the proband. This hypothesis is further supported by the evidence that a single gene could be causing PCOS and alopecia [94]. Interestingly, evidence from the studies in small cohorts of mono- and dizygotic twin pairs suggested that PCOS is an X-linked polygenic disorder [95,96]. Moreover, another report estimated that the risk of developing PCOS due to the family inheritance of the PCOS-causing gene was as high as 72% [97]. Similarly, our study also found that medical students in the study with PCOS had a previous family history of PCOS. In a study, 2.9% of medical students reported the presence of PCOS in their families. Another study also reported that 12.6% of the medical students with PCOS had their mother or at least one of their sisters displaying similar symptoms [41]. Other risk factors in the family history of medical students were diabetes, CVDs, menstrual irregularities, and cancer as found by our studies.

Women are typically seen as the main driving force within a family and in the community. The physical and mental discomfort associated with PCOS will typically lead to a reduced QOL for more than just the woman affected. Therefore, a healthy lifestyle and adequate knowledge of PCOS among women from an early age would be beneficial for society. Unfortunately, studies show that young women are typically not aware of the severity of PCOS [16]. Our study finds inadequate knowledge and awareness among medical and dental students about PCOS. For example, medical students knew the term PCOS, but 61.9% were unaware of how it develops [15]. In the same study, 71.7% of the students believed that irregular menstrual cycles can be the most common symptom of PCOS. One research study suggested that 42.2% of the students gained knowledge of the disease from social media and the internet [41]. The knowledge gap about PCOS leads to inappropriate lifestyle behavior, which may further predispose women to PCOS [98]. Indeed, one of the reports included in our study found that 19% of students lacked physical exercise, 81% of students with PCOS consumed meat daily or more than once a day in their diet, and 30% consumed vegetables only once a week [42]. The same study also found very frequent use of fast food among students with PCOS. Delayed diagnosis due to a lack of knowledge has become an important hurdle for the management and treatment of PCOS [98,99]. Another research study reported that 66.3% of the women with PCOS had incomplete knowledge about the risks of PCOS due to a lack of open discussions on female reproductive health in school curriculum and among family members [100]. Generally, it is observed that with higher learning, students gain knowledge of PCOS either through their peers or through the many educational lectures [101].

Finally, our studies also explored the interventions adopted by the students to alleviate PCOS symptoms. Since women with PCOS experience, several complications affecting multiorgan systems, an individualized treatment plan based on the presentation of the patient's symptoms seems appropriate. Metformin has become a preferred pharmacological treatment among non-obese women with PCOS to assist with anovulatory menstruation and thus, provides an advantage over other drugs in preventing infertility due to PCOS [102]. Hormonal contraceptives are also used in combination with metformin for irregular menses and dermatologic manifestations [103]. Similarly, our study found that among those diagnosed with PCOS, 29.1% were on oral contraceptive pills, 6.6% were on metformin alone, and 25% were on a combination of both drugs [39]. Interestingly, 29.1% in the same study were on no medications. Another study found that out of 47 medical students with PCOS, 76.6% were not taking any pharmacological treatment, which is alarming. Recent evidence suggests that women with PCOS should use hormonal contraceptives containing low ethynyl estradiol doses as these do not affect carbohydrate metabolism or pose the risk of developing type-2 diabetes mellites. Whereas progestogen-only contraceptives should be used in the presence of associated chronic vasculopathy as recommended for a long history of diabetes [103]. Therefore, proper clinical guidance on therapeutic intervention type and duration of the pharmacological treatment will be effective in the management of PCOS among medical students.

PCOS and the QOL

PCOS in women affects the general health-related QOL (HRQOL) and mental health as well [104,105]. Chronic and undiagnosed PCOS from a young age leads to lifelong disease burden across the world [106]. The adverse effects of PCOS pose a risk for long-term anxiety and other psychological conditions [107,108]. Most importantly, sociocultural perception and geographical regions play a role in psychological issues in PCOS [108,109]. Stress and associated mental issues may also lead to severe anxiety disorders and even suicidal tendencies [107,108]. Medical and dental students worldwide have already been found to have higher levels of perceived stress and emotional distress. Therefore, menstrual abnormalities and the associated symptoms of PCOS may lead to class absenteeism and diminished social life, which further add to the high-stress levels due to the inability of the students to prepare for exams, especially for the challenging medical curriculum [110]. Overall, our paper sheds light on the impact of PCOS, its risk factors, and the current trends in intervention towards the academic and personal qualities of the lives of female medical and dental students. A proactive approach by medical educators to foster an awareness of PCOS will reduce the knowledge gap among the students leading to adopt better lifestyle choices in the prevention and management of PCOS. Moreover, providing additional resources to accommodate the needs of students with PCOS in the medical institutions would be a fair and just treatment in achieving equality among everyone.

Strengths and limitations

This study is the first systematic review and meta-analysis to provide a comprehensive picture of PCOS among female medical and dental students in the last three years of research. It would add to the existing body of growing literature focusing on female physical and emotional well-being. This study should also encourage our medical learners and educators to adopt appropriate lifestyle behavior for good health and QOL. The present study, however, did not find relevant research from the USA, UK, and Australia within our objectives. Although the present study provided a comprehensive search strategy from the major databases, a few studies may have been overlooked, which would be unintentional. The exclusion of gray may also have excluded valuable information regarding our aims and objectives. The study found a significant heterogeneity in the prevalence of PCOS, which could be due to the diversity in population, locations, and methodologies to conduct the various studies. The high heterogeneity of results could be resolved in the future by introducing more subgroup analysis with more population data. Despite the limitations of the study, it provides valuable information that could be adopted for educational reformation to accommodate the needs of students with PCOS.

## Conclusions

PCOS is a silent disorder that has become a serious threat to the physical and mental well-being of women’s health. Early diagnosis leads to better management of the clinical features associated with PCOS. However, there are differing published scales used to evaluate the symptoms of PCOS leading to the variability in prevalence statistics worldwide. Considering the diverse etiology of the pathogenesis of PCOS including insulin resistance, adipogenicity, and androgenicity, patients with significant family history should also be evaluated for genetic and environmental factors. Further research on diverse populations may open new avenues to identify the candidate genes for targeted therapy. Metabolic and system-specific complications should also be considered and followed medically to avoid long-term complications such as endometrial cancer. Increased awareness of PCOS-associated comorbidities among young populations such as medical students would help in the early detection and management of PCOS. It will be interesting to compare the prevalence of PCOS and associated factors among female medical students in USA, UK, and Australia with the rest of the world in a large-scale study to find out the personal, professional, and economic impact of the disease. These studies will lead to improved diagnosis for women. With strong statistical studies using a comprehensive and globally accepted evaluation scale, the true prevalence of PCOS can be determined. This in turn may lead to a worldwide understanding of the difficulties experienced by women with PCOS. This information could be the driving force in facilitating changes in the medical school curriculum that has stringent attendance policies for academic events and exam writing with little consideration for people who suffer from cyclic rather than chronic disorders. A tailor-made structured program geared towards the psychological well-being of students should be implemented alongside the traditional curriculum. This could help create awareness, reduce anxiety, and improve the overall social quality of a student’s life.
